# Adsorption of surface functionalized silica nanoparticles onto mineral surfaces and decane/water interface

**DOI:** 10.1007/s11051-012-1246-1

**Published:** 2012-10-30

**Authors:** Cigdem O. Metin, Jimmie R. Baran, Quoc P. Nguyen

**Affiliations:** 1 Department of Petroleum and Geosystems Engineering, The University of Texas at Austin, 200 E. Dean Keeton, Stop C300, Austin, TX 78712 USA; 2 3M Corporate Research Materials Laboratory, 3M Center, Building 0201-01-W-28, St. Paul, MN 55144-1000 USA

**Keywords:** Silica nanoparticles, Surface-modified silica nanoparticles, Contact angle, Adsorption of nanoparticles, Interfacial tension

## Abstract

The adsorption of silica nanoparticles onto representative mineral surfaces and at the decane/water interface was studied. The effects of particle size (the mean diameters from 5 to 75 nm), concentration and surface type on the adsorption were studied in detail. Silica nanoparticles with four different surfaces [unmodified, surface modified with anionic (sulfonate), cationic (quaternary ammonium (quat)) or nonionic (polyethylene glycol (PEG)) surfactant] were used. The zeta potential of these silica nanoparticles ranges from −79.8 to 15.3 mV. The shape of silica particles examined by a Hitachi-S5500 scanning transmission electron microscope (STEM) is quite spherical. The adsorption of all the nanoparticles (unmodified or surface modified) on quartz and calcite surfaces was found to be insignificant. We used interfacial tension (IFT) measurements to investigate the adsorption of silica nanoparticles at the decane/water interface. Unmodified nanoparticles or surface modified ones with sulfonate or quat do not significantly affect the IFT of the decane/water interface. It also does not appear that the particle size or concentration influences the IFT. However, the presence of PEG as a surface modifying material significantly reduces the IFT. The PEG surface modifier alone in an aqueous solution, without the nanoparticles, yields the same IFT reduction for an equivalent PEG concentration as that used for modifying the surface of nanoparticles. Contact angle measurements of a decane droplet on quartz or calcite plate immersed in water (or aqueous nanoparticle dispersion) showed a slight change in the contact angle in the presence of the studied nanoparticles. The results of contact angle measurements are in good agreement with experiments of adsorption of nanoparticles on mineral surfaces or decane/water interface. This study brings new insights into the understanding and modeling of the adsorption of surface-modified silica nanoparticles onto mineral surfaces and water/decane interface.

## Introduction

Nanoparticles have shown promise in many potential applications for the characterization and production of hydrocarbon producing formations (Mokhatab et al. 2006). The use of nanoparticles as sensors (Prodanovic et al. 2010), enhanced oil recovery (EOR) agents (Holcomb 2012; Moon 2008) or drilling fluid additives (Cai et al. 2011) are among the main topics of research. In the process of designing nanoparticles to be used as sensors or EOR agents, the retention of nanoparticles due to the adsorption onto minerals and/or at the water/oil interface is the fundamental issue. The degree of adsorption would determine the extent of contact angle change (wettability alteration) and/or decrease in interfacial tension (IFT). Therefore, it plays an important role in choosing the types of nanoparticles or surface modifying agents for said nanoparticles.

Silica nanoparticles are good candidates for such applications due to their low cost of fabrication, their ready availability, and the ability to modify their surfaces by known chemical methods. The surface modification of silica nanoparticles would allow one to control their hydrophilicity and also to improve their salt tolerance. There exists a critical salt concentration (CSC) below which previously studied silica nanoparticles stayed well dispersed in water (Metin et al. 2011). The surface modification significantly improves CSC especially for divalent cations (Ca^2+^ and Mg^2+^). Therefore, they can be injected in reservoir rocks where brine salinity is large and remain as a stable dispersion.

The interaction of nanoparticles with liquids (water/oil interface) or solids (mineral surfaces) determines the mechanisms of retention of nanoparticles in reservoir rocks. Characterization of the surface charge of nanoparticles by measuring their zeta potential, tracking nanoparticles in the bulk phase or at interface by UV–Visible spectroscopy provided means to analyze the effect of pH, surface modification of nanoparticles and their sizes on the stability of nanoparticles at fluid interfaces. A comprehensive literature review on nanoparticles at fluid interfaces is presented by Bresme and Oettel (2007).

Lin et al. (2005) presented an experimental study on the structure of hydrophobically surface-modified 4.6 nm cadmium selenide nanoparticle assembly at fluid interfaces. They observed that nanoparticles assembled at the interface of two immiscible liquids (toluene and water) as a densely packed monolayer. In the case of particles with different sizes, larger particles displaced smaller particles at a rate consistent with their adsorption energy. The assembly at the water/toluene interface was liquid-like with no long-range order. Lee et al. (2006) studied the monolayer behavior of 500 nm silica particles in the presence of a cationic surfactant at the air/water interface. They compared chemically grafted and physically modified nanoparticles and found that modification methods and chain length of modifying agents determined the structure of particle layering at the interface.

Reincke et al. (2006) discussed three types of interactions that are dominant for a charged nanoparticle (less than 16 nm gold nanoparticles) at a water/oil interface: energy of water/organic, water/particle and particle/organic interfaces, electrostatic repulsion between particles and van der Waals interactions between particles at the interface. They reported that big particles adsorbed more strongly to the interface than small particles. Binks and Fletcher (2001) studied the theoretical adsorption of amphiphilic spherical particles (Janus particles) at the oil/water interface. Later, Binks and Whitby (2005) found that precipitated silica particles with a primary particle size ranging from 3.5 to 101 nm could stabilize oil-in-water emulsions. The emulsion stability was controlled by changing the pH or particle charge. The authors observed that adding cationic surfactants improved the emulsion stability. The average diameter of emulsions increased as the silica nanoparticle size increased. Bresme and Quirke (1999) analyzed the wetting behavior of spherical particles at liquid/water interface by using MD simulation. Young’s equation provided an accurate estimation of the contact angle even for particle of size 1.5 nm. Contact line tension appeared to have no effect on the contact angle when the surface tensions were on the order of that of water.

Particle structuring in a wedge film and the role of structural component of disjoining pressure on displacement of the contact line were studied by Wasan and Nikolov (2003).

The authors observed by video microscopy, a crystal like ordering of 1-μm diameter latex particles in the liquid film-meniscus region of wedge-like shape, which resulted in a structural component of disjoining pressure. Then, the authors argued that the structural component of the disjoining pressure was strong enough for a nanofluid composed of 8-nm diameter micelles to move the contact line at oil droplet/glass/aqueous micellar solution interface. This particle structure formation in the wedge film was confirmed by the theoretical results of Boda et al. (1999). Further theoretical studies followed their research (Chengara et al. 2004; Vafaei et al. 2006; Matar et al. 2007; Sefiane et al. 2008). However, it is not clear that the structural disjoining pressure is the only mechanism influencing this enhanced spreading of a droplet in the presence of nanoparticle suspensions. Vafaei et al. (2006) conducted contact angle measurements of droplets containing 2.5 nm bismuth telluride nanoparticles, which are surface-modified with thioglycolic acid, on glass and silicon wafer substrates in air. The authors observed that the variation in contact angle depended on the solid surface material and nanoparticle size. At a given concentration, smaller diameter nanoparticles resulted in greater changes in contact angle than larger diameter nanoparticles would. The authors argued that greater amount of smaller diameter nanoparticles can fit into this region than larger diameter ones. The spreading of a sessile droplet on solid surface was also studied theoretically by Yang et al. (1991), Blake et al. (1997), de Ruijter et al. (1999a, b), Hwang et al. (2001), Choi and Kim (2006) and Voronov et al. (2006, 2007).

In this study, we investigate the interaction of unmodified or surface-modified silica nanoparticles with mineral surfaces and decane/water interface. We carried out adsorption experiments with the silica nanoparticles onto quartz and calcite surfaces. IFT measurements provide insightful information on the interaction of silica nanoparticles with decane/water interface. The effects of particle size, concentration and surface type of silica nanoparticles are also studied in detail. We highlight the importance of surface modifiers on silica nanoparticles and the design of experiments when studying the adsorption of nanoparticles with minerals or water/hydrocarbon interface. Contact angle measurements confirm our findings from nanoparticle dispersion/mineral and nanoparticle dispersion/decane interactions.

## Materials and methods

The materials studied were aqueous dispersions of silica particles as provided by 3 M, Co (St. Paul, MN, USA). The mean diameters of primary particles are 5, 25, and 75 nm, which have an unmodified surface or a modified surface with sulfonate, PEG or quaternary ammonium and PEG. The latter one will be referred to as “quat” throughout this article. The surface modifications describe the surface of the particles after using alkoxysilanes as surface modifying agents. The zeta potential of these silica nanoparticles was determined using a Malvern Zetasizer. The values are presented in Table 1. We used Iceland spar calcite and Ottowa quartz sand for these studies. The zeta potential of the mineral powders were also measured using the Zetasizer and were found to be −55 mV for quartz and −31 mV for calcite.

**Table 1:** Zeta potential of silica nanoparticles dispersed in water

Particle diameter (nm)	Surface type	Zeta potential (mV)
5	PEG	−24.1
25	PEG	−39.3
75	PEG	−50.0
5	Sulfonate	−31.3
25	Sulfonate	−44.2
75	Sulfonate	−52.8
5	Quat	9.3
25	Quat	−1.1
75	Quat	15.2
5	Unmodified	−48.7
25	Unmodified	−60.3
75	Unmodified	−79.8

We used a Cary 50 ultraviolet–visible spectrophotometer (UV–Vis) to determine the concentration of nanoparticles in the supernatant liquid. IFT measurements were measured using a Kruss K100 tensiometer equipped with a Wilhemly plate. A Rame-Hart contact angle goniometer was used to determine the contact angle of decane droplets on mineral samples immersed in water or nanoparticle dispersion.

Pieces of minerals were submersed in the various nanoparticle dispersions for 24 h. The liquid of an amount of 3 ml was separated from the mineral by pipette and centrifuge. The supernatant liquid was then analyzed by UV–Vis spectroscopy to determine the silica nanoparticle concentration remaining in the liquid. Principal component analysis combined with multiple regression was applied to construct calibration curves for the particle concentration analysis using the Unscrambler chemometric software. The supernatant liquid was centrifuged at 14,000 rpm for 40 min to separate fines generated by the mineral grains. The nanoparticle dispersions of 0.04, 0.2, and 1 wt% were added to mineral to 10:1 and 5:2 dispersion to mineral weight ratios. These liquid-to-solid ratios were chosen based on the range of the ratios commonly used during sorption experiments published in literature (Antelmi and Spalla 1999; Marczewski and Szymula 2002; Flury et al. 2004).

The calcite mineral was first ground using an agate mortar and pestle set and sieved using meshed sieves ranging from 20 to 100 mesh for 20 min under the agitation of a Ro-Tap sieve shaker. The grains were then cleaned by deionized (DI) water before the adsorption tests. The UV–Vis absorbance of the supernatant was measured as a part of the cleaning procedure to make sure that the substrate was cleaned with DI water. Then the clean calcite grains were air dried at room temperature. The same cleaning procedure was applied to the quartz sand. We use 20/35 (841/500 μm) mesh calcite and 20/40 (841/420 μm) mesh quartz sand. To study the effect of mineral size we also choose 60/100 (250/150 μm) mesh calcite and quartz sand.

The surface energy of clean and dry quartz sand and calcite grains were measured using an inverse gas chromatography (IGC) method. IGC involves the sorption of a known adsorbate (vapor) and an unknown adsorbent stationary phase (solid sample). The principle of this method has been described in detail elsewhere (Saint Flour and Papirer 1982). The experimental procedure can be briefly described as follows. The series of alkanes used for determining the dispersive surface energy were obtained from Acros Organics and were of the High Performance Liquid Chromatography (HPLC) grade. The cleaned calcite or quartz samples were then packed into the column and flushed with the carrier gas, He at 105 °C for 2 h to remove any trace of moisture contamination. The column is then conditioned for another 2 h by passing the carrier gas, helium at the desired temperature and relative humidity. The possibility of any moisture accumulation is removed because of continuous outgassing of the column first at elevated temperature and then at the desired temperature. Then a series of solvent pulse injections are carried out and their retention behavior monitored by the Flame Ionization Detector (FID) and Thermal Conductivity Detector (TCD) placed at the end of the column. The retention times are recorded and used to determine the total surface energy of the quartz and calcite samples (Saint Flour and Papirer 1982).

## Results and discussions

### Adsorption on minerals

**Fig. 1:**
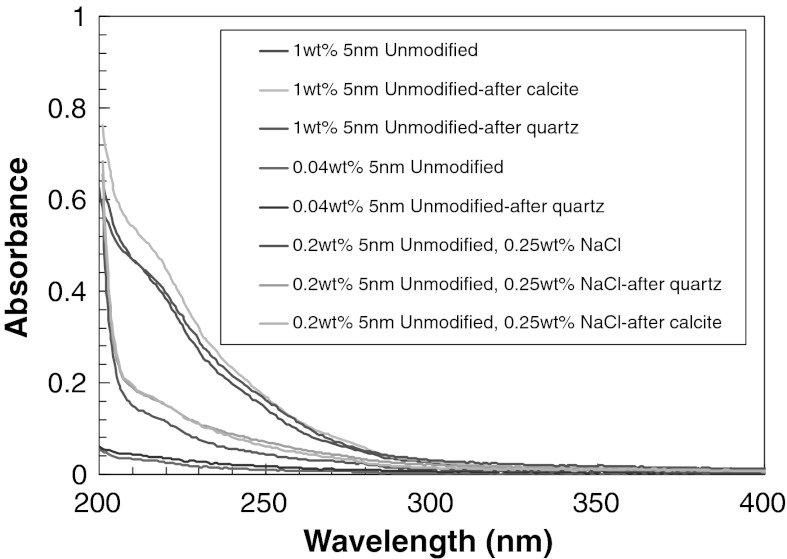
UV–Vis spectra of 0.04, 0.2, and 1 wt% 5 nm unmodified silica nanoparticle dispersion with and without NaCl before and after contact with quartz sand or calcite grains

The batch adsorption experiments were carried out with 150 and 500 μm calcite grains using silica nanoparticle concentrations of 0.04, 0.2, and 1.0 wt%. The UV–Vis spectra of the 5 nm unmodified silica nanoparticle dispersions are presented in Fig. 1 before and after contact with calcite grains. For the silica concentrations studied (0.04, 0.2, and 1 wt%) there is no significant adsorption of nanoparticles on calcite surfaces. The effect of grain size was studied with 60/100 mesh calcite and no significant adsorption is observed.

**Fig. 2:**
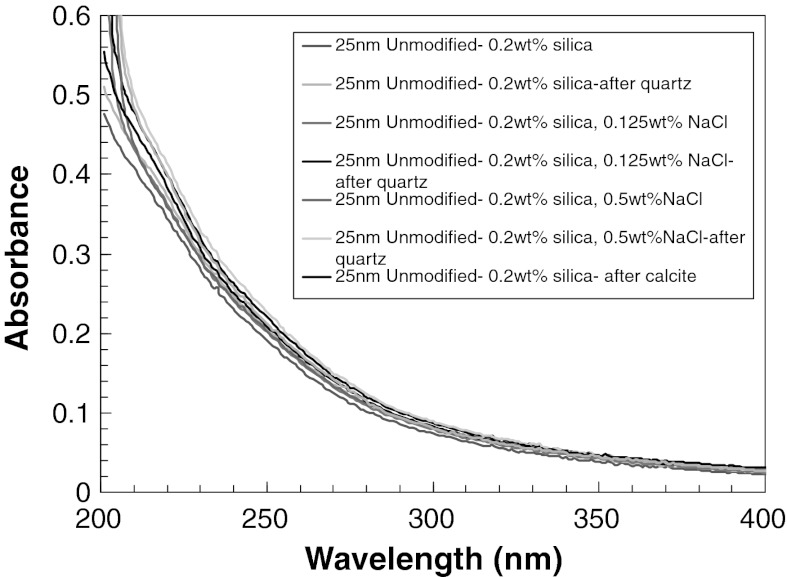
UV–Vis spectra of 0.2 wt% 25 nm unmodified silica nanoparticle dispersion with or without NaCl before and after contact with quartz sand or calcite grains

The effect of electrolyte on adsorption of silica nanoparticles onto a calcite surface was tested by adding 0.25 wt% NaCl to 0.2 wt% unmodified silica nanoparticle dispersion. The NaCl concentration is below CSC at 0.5 wt% (CSC) (Metin et al. 2011) to ensure that the nanoparticle dispersion is stable. Figure 2 shows that there is no significant adsorption in the presence of NaCl. Moreover, increasing the size of nanoparticles (25 nm diameter) does not influence the adsorption of unmodified silica nanoparticles on calcite surface (Fig. 2).

**Fig. 3:**
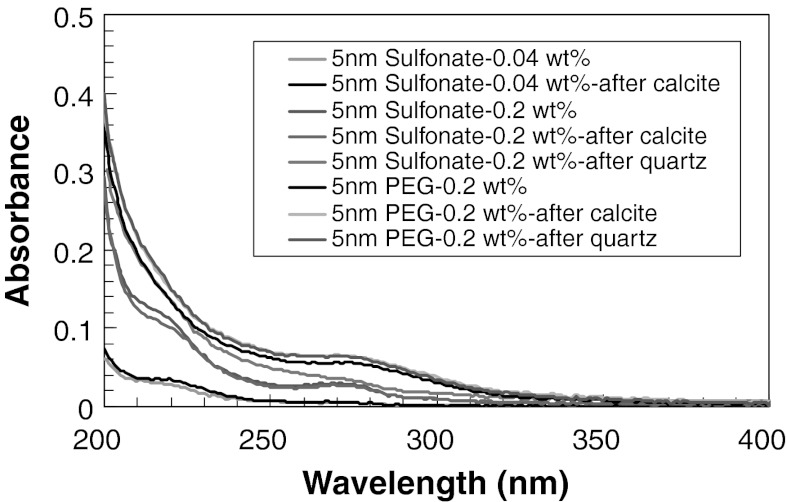
UV–Vis spectra of 0.04 and 0.2 wt% 5 nm sulfonate or PEG-modified silica nanoparticle dispersion after contact with quartz sand or calcite grains

We also studied the effect of the surface modification of silica nanoparticles on the adsorption behavior. The results show that there was no significant adsorption of PEG- or sulfonate-modified nanoparticles on calcite (Fig. 3).

The adsorption of silica nanoparticles onto quartz sand was studied with batch adsorption experiments. From Fig. 1, we concluded that there is no significant adsorption of unmodified silica nanoparticles onto quartz surface. The effect of grain size was studied with 60/100 mesh quartz sand and no significant adsorption was observed. Figure 2 shows that at 0.5 wt% NaCl concentration, there was not any significant adsorption of silica nanoparticles on quartz surface.

The effect of the surface treatment is presented in Fig. 3 for sulfonate- and PEG-modified particles, respectively. Similar to the observations with unmodified particles there was not any significant adsorption of surface-modified particles on quartz sand.

DLVO (Derjaguin and Landau 1941; Verwey and Overbeek 1948) theory was used to model the particle–mineral interactions and compare those results to the experimental results. The electrostatic repulsion energy can be expressed for two parallel, infinite plates with flat double layers as.

1 $$ V_{\rm R} = \frac{\varepsilon \kappa }{8\pi }\left[ {\left( {\psi_{1}^{2} + \psi_{2}^{2} } \right)\left( {1 - \coth \kappa h} \right) + 2\psi_{1} \psi_{2} \cos ech(\kappa h)} \right], $$

where $\psi_{1}$ and $\psi_{2}$ are the surface potential of plates 1 and 2, *κ* is the inverse of electrical double layer, and *h* is the separation distance. For two spherical colloidal particles, Derjaguin approximation for $\kappa a \gg 1$ gives

2 $$ V_{\text{R}} = \frac{{\varepsilon a_{1} a_{2} \left( {\psi_{1}^{2} + \psi_{2}^{2} } \right)}}{{4(a_{1} + a_{2} )}}\left[ {\frac{{2\psi_{1} \psi_{2} }}{{\left( {\psi_{1}^{2} + \psi_{2}^{2} } \right)}}\ln \left( {\frac{1 + \exp ( - \kappa h)}{1 - \exp ( - \kappa h)}} \right) + \ln \left( {1 - \exp ( - 2\kappa h)} \right)} \right], $$

where *a*_1_ and *a*_2_ are the radii of particles. Hogg et al. (1966) showed that Debye–Huckel approximation works well even at large surface potentials for *h* > *a*.

Thin, slightly overlapping cloud of a spherical particle and a flat plate gives a repulsive energy approximated by Eq. 3.

3 $$ V_{\rm R} = 16\varepsilon a\left( \frac{kT}{ze} \right)^{2} \tanh \left( {\frac{{ze\psi_{\rm s} }}{4kT}} \right)\tanh \left( {\frac{{ze\psi_{\rm p} }}{4kT}} \right)\exp \left( { - \kappa h} \right), $$

where subscripts s and p represent the spherical particle and the flat plate, respectively. Derjaguin’s approximation is valid for all values of surface potentials provided that *κa* ≫ *κh* ≫ 1.

The van der Waals attraction potential between two spheres of radii *a*_1_ and *a*_2_ is given in Eq. 4.

4 $$ \begin{aligned} V_{\text{A}} & = & - \frac{{A_{132} }}{6}\left[ {\frac{{2a_{1} a_{2} }}{{R^{2} - (a_{1} + a_{2} )^{2} }} + \frac{{2a_{1} a_{2} }}{{R^{2} - (a_{1} - a_{2} )^{2} }} + \ln \left( {\frac{{R^{2} - (a_{1} + a_{2} )^{2} }}{{R^{2} - (a_{1} - a_{2} )^{2} }}} \right)} \right] \\ R & = & a_{1} + a_{2} + h \\ A_{132} & = & \left( {\sqrt {A_{11} } - \sqrt {A_{33} } } \right)\left( {\sqrt {A_{22} } - \sqrt {A_{33} } } \right) \\ \end{aligned} ,$$

where *A*_132_, the Hamaker constant of silica nanoparticle (1), water (3) and mineral (2) is calculated from the measured dispersive surface energies of calcite (71.76 mJ/m^2^) and quartz (107.78 mJ/m^2^). The results of *A*_*132*_ for calcite and quartz are calculated as 1.09 × 10^−20^ and 1.62 × 10^−20^ J, respectively.

Similarly, the van der Waals attraction between sphere and a planar half-space plate can be expressed as

5 $$ V_{\text{A}} = - \frac{{A_{132} a}}{6h}\left[ {1 + \frac{h}{2a + h} + \frac{h}{a}\ln \left( {\frac{h}{2a + h}} \right)} \right] $$

For details of above equations, please see Hunter (2001), Goodwin (2009) and Hogg et al. (1966).

**Fig. 4:**
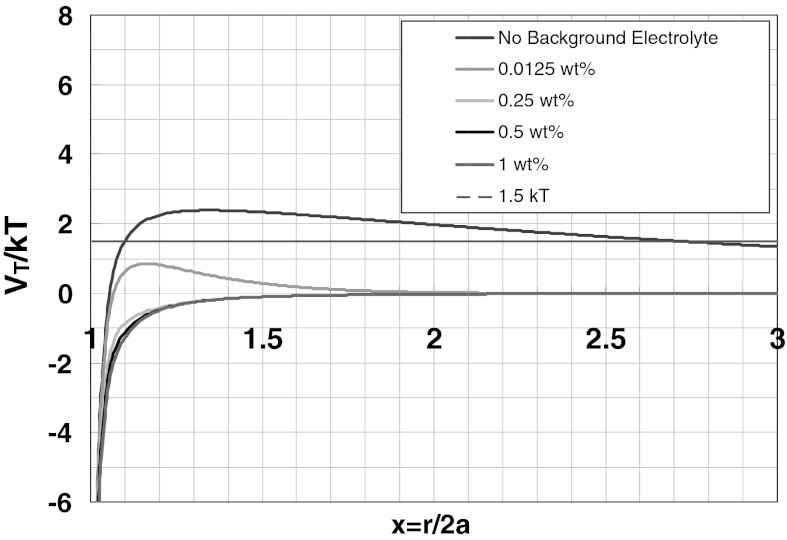
Total interaction energy between calcite plate and 25-nm diameter silica nanoparticles as a function of NaCl concentration (Eqs. 3, 5)

The total interaction potential *V*_T_ = *V*_A_ + *V*_R_ is calculated for the 25 nm unmodified silica nanoparticles–calcite interaction by using Eqs. 3 and 5. The results are shown in Fig. 4 at various NaCl concentrations. Although the energy barrier is small, the predictions by DLVO indicate that there is no adsorption without background NaCl concentration. However, at 0.5 wt% NaCl concentration the interaction between the silica nanoparticle and the calcite grain is attractive. This prediction does not agree with the experimental results as shown in Fig. 2. The interaction potential by DLVO theory was also calculated for 5 nm unmodified silica nanoparticle–calcite interaction. (Note that the condition, *κa* ≫ 1, in the approximation of repulsive energy is not satisfied for these small size nanoparticles). The interaction energy is repulsive in the absence of background NaCl concentration, however, the magnitude of the energy barrier is also small which can be easily overcome by the kinetic energy of particles in dispersion.

**Fig. 5:**
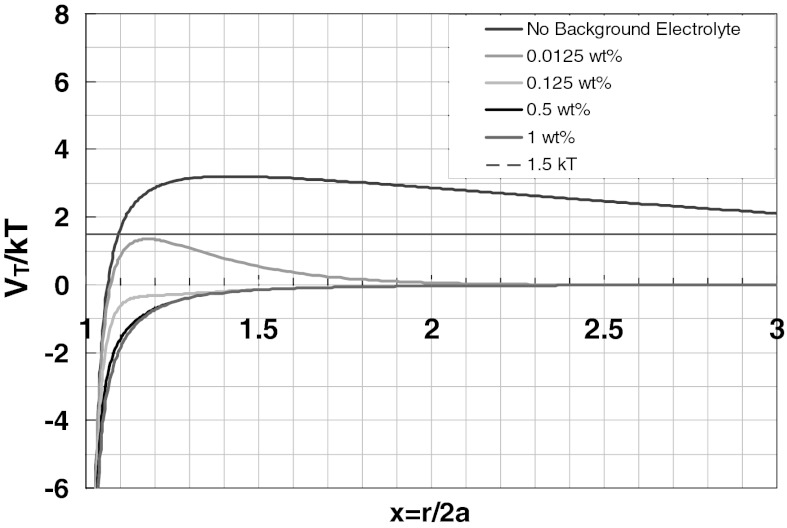
Total interaction energy between 420-μm diameter quartz grain and 25-nm diameter silica nanoparticles as a function of NaCl concentration (Eqs. 2, 4)

Similar results in DLVO curves are obtained for 25 nm unmodified silica nanoparticles–quartz interaction potential by using Eqs. 2 and 4. Experimental results shown in Fig. 2 agree well with DLVO predictions (Fig. 5) for the condition where there is no background electrolyte, but we did not observe any significant adsorption at 0.5 wt% NaCl as predicted by DLVO. For 5 nm unmodified silica nanoparticles–quartz interaction potential the particle size is too small to satisfy the condition *κa* ≫ 1. A small energy barrier occurs which would be overcome by silica nanoparticles promoting the adsorption on quartz. However, insignificant adsorption is experimentally observed.

### IFT of silica nanoparticle dispersion/decane interface

**Fig. 6:**
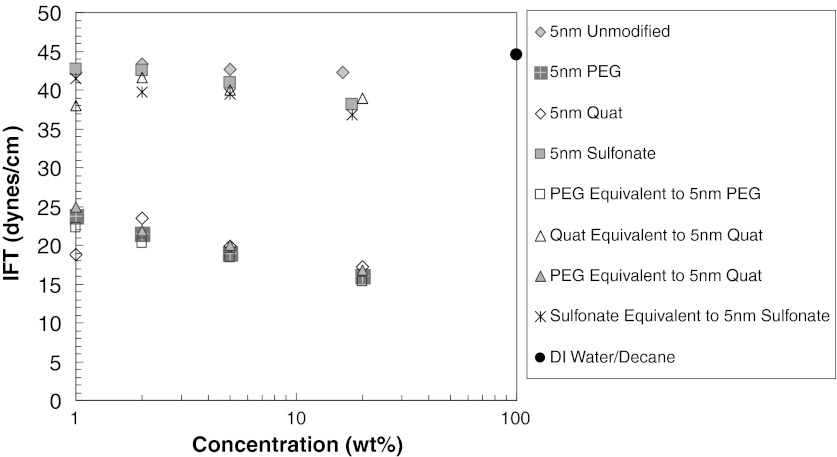
IFT of decane/water in presence of 5 nm silica unmodified or surface-modified nanoparticles

**Fig. 7:**
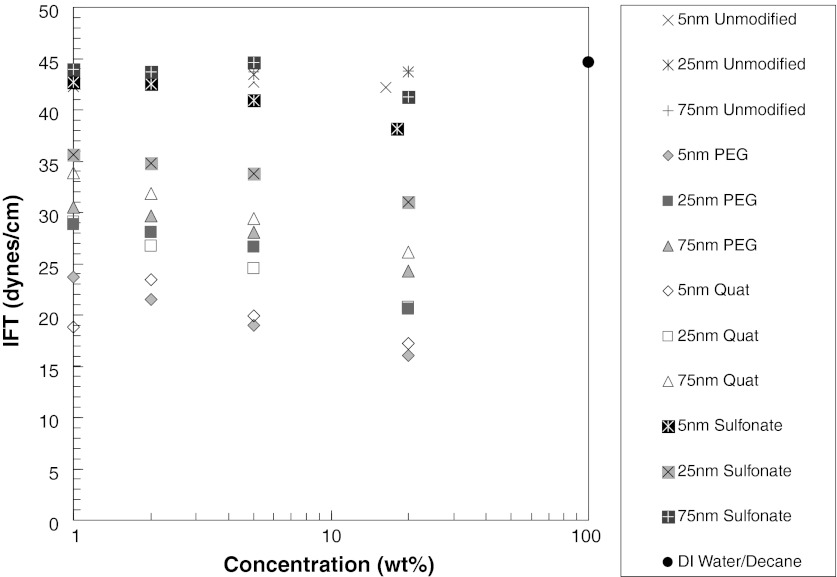
IFT of decane/water in presence of nanoparticles. The effect of nanoparticle size and concentration is shown

The effects of nanoparticles on interfacial properties are investigated with unmodified and surface-modified silica nanoparticle dispersions. The Wilhemy plate method with a Kruss K100 tensiometer was used to determine the effect of nanoparticles on the IFT of decane/water interface. The results are presented in Figs. 6 and 7. The IFT of decane/water is 45 dynes/cm. Unmodified silica nanoparticles at various concentrations do not have any effect on IFT of water/decane interface (43 dynes/cm), as presented in Figs. 6 and 7. The surface-modified silica nanoparticles with sulfonate surface modification also do not influence the IFT either. A slight decrease is observed as particle concentration increases, but this decrease may be due to the presence of the sulfonate surface modifier. When the IFT in presence of sulfonate-modified particles is compared with just the sulfonate modifier in water, almost the same decrease in IFT is observed. Therefore, the decrease in IFT corresponds to the effect of sulfonate molecules not to the presence of the nanoparticles.

A significant decrease in IFT (24 dynes/cm) occurs with PEG-modified silica particles. To determine whether this decrease is because of the PEG itself or not, we prepared a solution having the same PEG concentration, but without nanoparticles. This PEG solution exhibits similar IFT values as the PEG-modified nanoparticle dispersions. Therefore, the presence of PEG, attached to silica nanoparticle or free in solution, determines the decrease in IFT of water/decane interface. The presence of the nanoparticle appears not to add to the IFT reduction.

The effect of particle size and concentration is presented in Fig. 7. The results are consistent with our findings for 5 nm particles. All the unmodified silica nanoparticle dispersions (5, 25, and 75 nm) have almost the same IFT value as water/decane, and it appears not to be sensitive to particle concentration or size. Based on these findings, it can be concluded that unmodified silica nanoparticles do not stay at the water/interface.

**Fig. 8:**
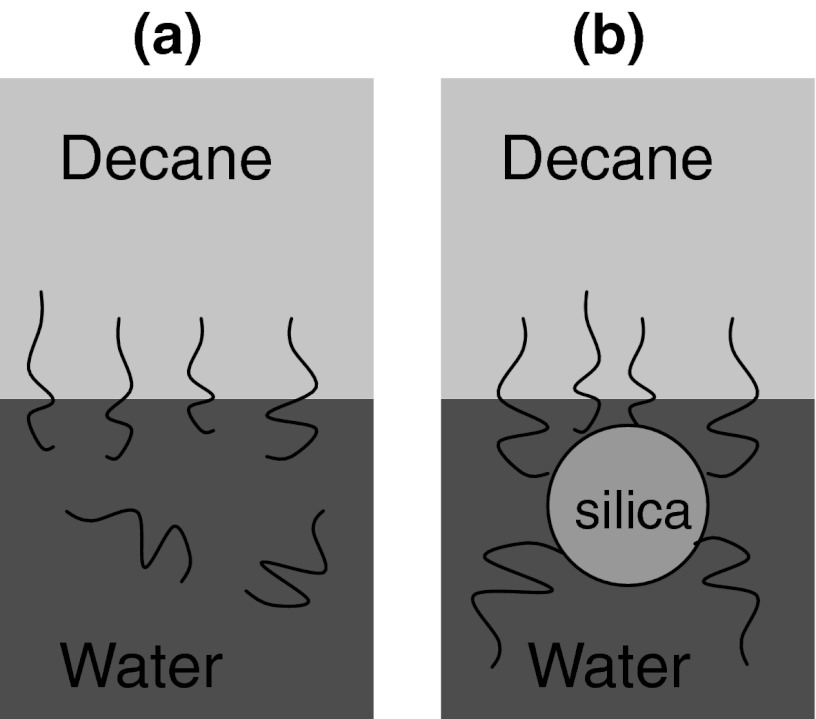
Schematic presentation of adsorption of PEG to water/decane interface **a** in the absence of silica nanoparticle and **b** attached to silica nanoparticle

However, with the surface-modified nanoparticles, a decrease in IFT is observed as particle concentration increases at a given size or as particle size decreases at a given nanoparticle concentration. These trends are consistent with the increasing amount of the surface modifiers as the nanoparticle concentration increases and the nanoparticle size decreases. In the case of surface-modified nanoparticles, deviations from IFT of water/decane occur, especially in case of PEG-modified silica nanoparticles, as seen in Fig. 8. The type and amount of surface treatment attached to silica nanoparticles determines the extent of the change in IFT of water/decane interface. The degree of IFT change is identical for aqueous solutions of surface modifying material in the absence of nanoparticles.

Insignificant adsorption of unmodified silica nanoparticles at the decane/water interface shows that the silica nanoparticles are not amphiphiles and the surface modification alone determines the adsorption of silica nanoparticles on interfaces as observed with the PEG-modified silica nanoparticles.

**Fig. 9:**
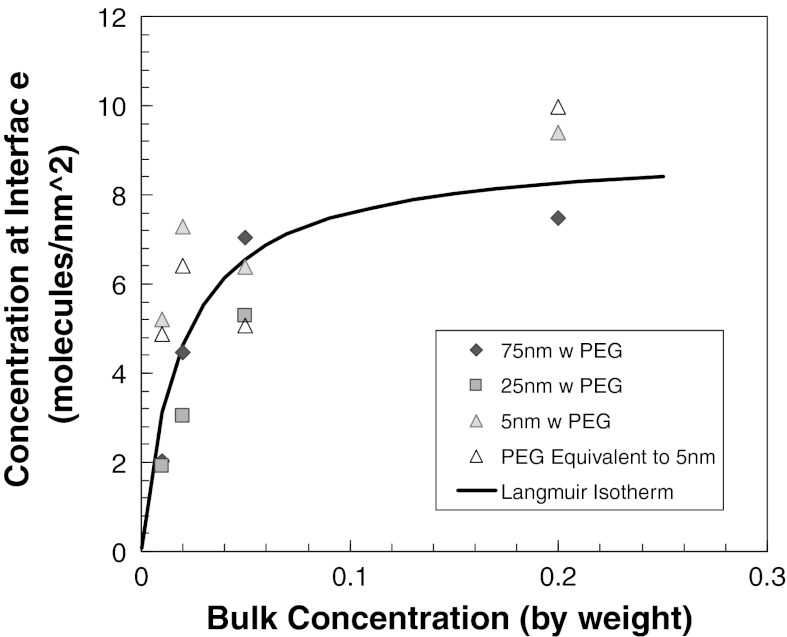
Adsorption of PEG or PEG-modified silica nanoparticles on decane/water interface

The concentration of PEG in aqueous solution and PEG attached to silica nanoparticles partitioned to the interface was quantitatively determined by using thermodynamic theory of partitioning (Gibbs equation):

6 $$ \frac{{{\text{d}}\gamma }}{{{\text{d}}C_{2} }} = - \Upgamma_{2} \frac{RT}{{C_{2} }}, $$

where *R* is the universal gas constant, *T* is the temperature, *C*_2_ is the bulk concentration, γ IFT, and Γ_2_ is the concentration at interface. The results are presented in Fig. 9. The line corresponds to Langmuir isotherm (Hunter 2001) in Eq. 7 that is used to fit our data. The model parameters, *K* and Γ_max_ are 52.8 and 9.04 (molecules/nm^2^), respectively.

7 $$ \Upgamma_{2} = \Upgamma_{\hbox{Max} } \frac{{KC_{2} }}{{1 + KC_{2} }} $$

### Contact angle measurements

**Fig. 10:**
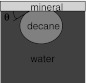
Schematic of an oil droplet on a solid substrate (mineral) immersed in water

The contact angle goniometer was used to monitor and measure the contact angle of decane droplet on quartz or calcite plate immersed in silica nanoparticle dispersions. The contact angle is measured through the denser phase (water or nanoparticle dispersion). A schematic is shown in Fig. 10. The contact angle θ = 0° corresponds to a surface completely water wet and θ = 180° corresponds to completely oil wet surface. For effective displacement of oil by water, we need θ < 90°.

**Fig. 11:**
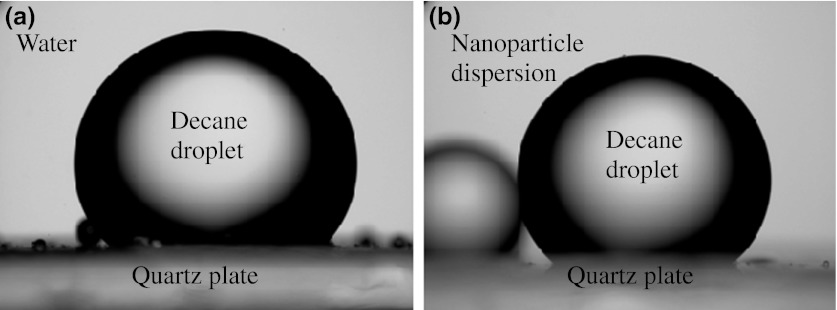
Decane droplet on quartz plate immersed in **a** water and **b** 5 nm unmodified silica nanoparticle dispersion of 1 wt%. The contact angle is **a** 59° and **b** 46°

**Fig. 12:**
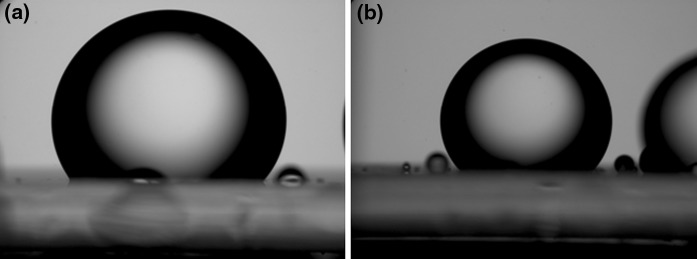
Decane droplet on quartz plate immersed in **a** water and **b** 25 nm unmodified silica nanoparticle dispersion of 1 wt%. The contact angle is **a** 60° and **b** 52°

**Fig. 13:**
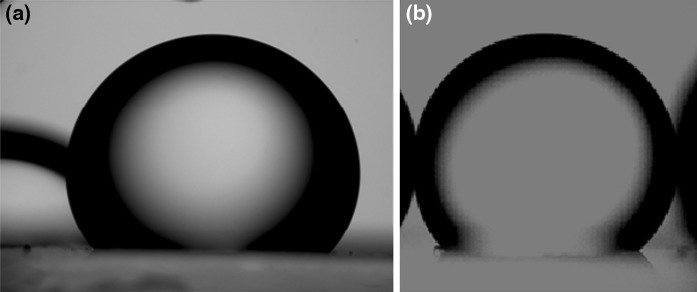
Decane droplet on quartz plate immersed in **a** water and **b** 75 nm unmodified silica nanoparticle dispersion of 0.5 wt%. The contact angle is **a** 56° and **b** 52°. The *color* in **b** is digitally altered to more easily see the droplet. (Color figure online)

Calcite and quartz plates were immersed in decane for 1 week before the contact angle experiments. The pictures of a decane droplet immersed in water or silica nanoparticle dispersion are presented in Figs. 11, 12, and 13. The effect of particle size and surface type on the contact angle of water/decane on a mineral was studied. The decane droplet is injected with an inverted J-syringe underneath the substrate. However, the pictures in Figs. 11, 12, and 13 are digitally inverted using a Pax-it 2+ digital camera connected directly to the microscope for visual purposes. These images are inverted pictures of the actual droplet.

The image on the left side in each figure corresponds to the contact angle of water/decane/mineral without nanoparticles and the image on the right side shows the contact angle at nanoparticle dispersion/decane/mineral. Figures 11, 12, and 13 show the effect of unmodified silica nanoparticles and their size on the contact angle on quartz plate. The contact angle does not change significantly in the presence of unmodified nanoparticles of 5, 25, or 75 nm diameter. This observation is consistent with the present findings of IFT and adsorption. The unmodified nanoparticles do not change IFT of water/decane nor do they adsorb to the quartz surface.

Figures 15, 16, 17 in the Appendix show the effect of sulfonate-modified silica nanoparticles and their size on the contact angle on quartz plate. The contact angle does not change significantly in the presence of sulfonate-modified nanoparticles of 5, 25, or 75 nm diameter. This observation is consistent with our findings of IFT and adsorption. The sulfonate-modified nanoparticles do not significantly change the IFT of water/decane interface nor do they adsorb to the quartz surface. Similar results for contact angle are observed with the quat-modified silica nanoparticles of 5, 25, or 75 nm diameter (Figs. 18, 19, 20 in Appendix).

Although PEG-modified nanoparticles reduce the IFT of water/decane from 45 to 24 dynes/cm there is no significant change in contact angle in the presence of these nanoparticles, under the experimental conditions. Figures 21, 22, and 23 in Appendix show the effect of PEG-modified silica nanoparticles and their size on the contact angle on quartz plate. The contact angle does not significantly change in the presence of PEG-modified nanoparticles of 5, 25 or 75 nm diameter. This observation is consistent with our findings from batch adsorption experiments. The effect of temperature is investigated with 5 nm PEG-modified nanoparticles at 80 °C, Fig. 24 in Appendix. We did not observe any significant change in the contact angle at the higher temperature.

Figures 25, 26, and 27 in Appendix show the effect of sulfonate-modified silica nanoparticles and their size on the contact angle on calcite plate. The contact angle does not significantly change in the presence of sulfonate-modified nanoparticles of 5, 25, or 75 nm diameter. This observation is also consistent with our findings of IFT and adsorption.

**Fig. 14:**
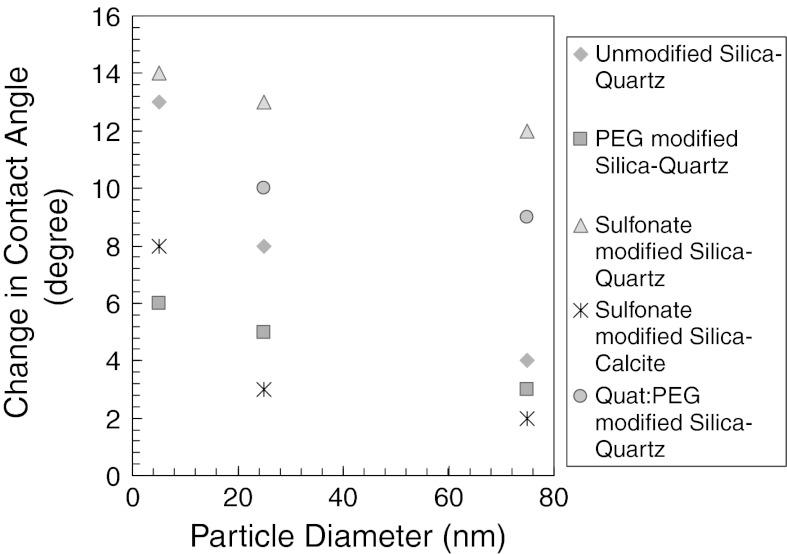
The change in contact angle in the presence of 1 wt% silica nanoparticles

**Fig. 15:**
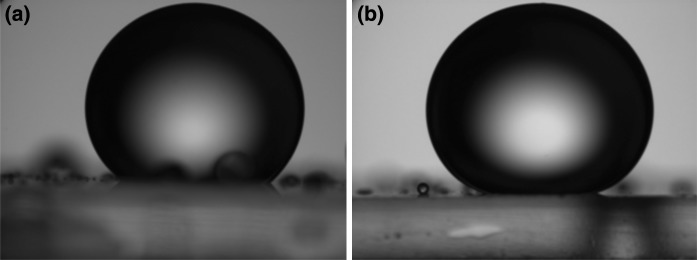
Decane droplet on quartz plate immersed in **a** water and **b** 5 nm sulfonate-modified silica nanoparticle dispersion of 1 wt%. The contact angle is **a** 34° and **b** 20°

**Fig. 16:**
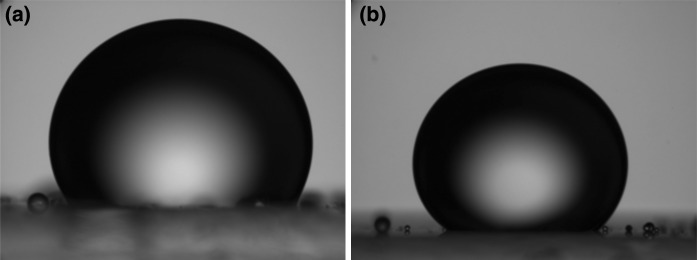
Decane droplet on quartz plate immersed in **a** water and **b** 25 nm sulfonate-modified silica nanoparticle dispersion of 1 wt%. The contact angle is **a** 51° and **b** 38°

**Fig. 17:**
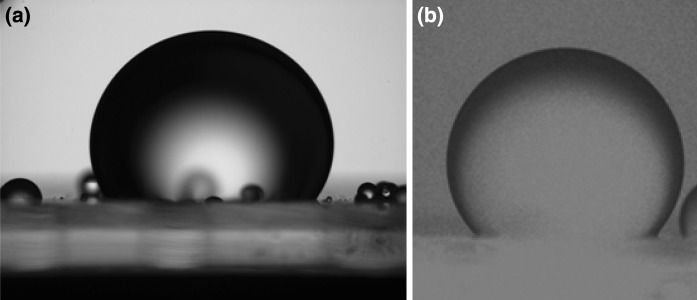
Decane droplet on quartz plate immersed in **a** water and **b** 75 nm sulfonate-modified silica nanoparticle dispersion of 0.5 wt%. The contact angle is **a** 55° and **b** 43°. The *color* in **b** is digitally altered to see the droplet clearly since the light transmittance of nanoparticle dispersion is low because of particle size. (Color figure online)

**Fig. 18:**
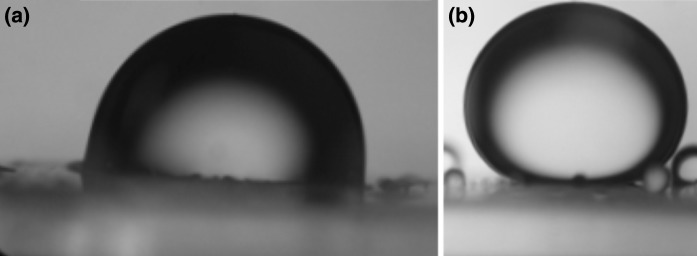
Decane droplet on quartz plate immersed in **a** water and **b** 5 nm quat/PEG (50:50)-modified silica nanoparticle dispersion of 1 wt%. The contact angle is **a** 91° and **b** 14°

**Fig. 19:**
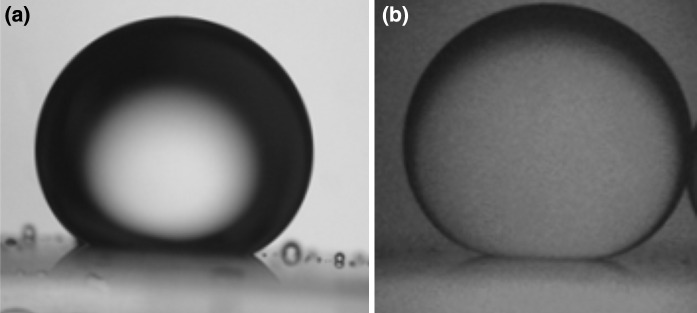
Decane droplet on quartz plate immersed in **a** water and **b** 25 nm quat/PEG (50:50)-modified silica nanoparticle dispersion of 1 wt%. The contact angle is **a** 30° and **b** 20°

**Fig. 20:**
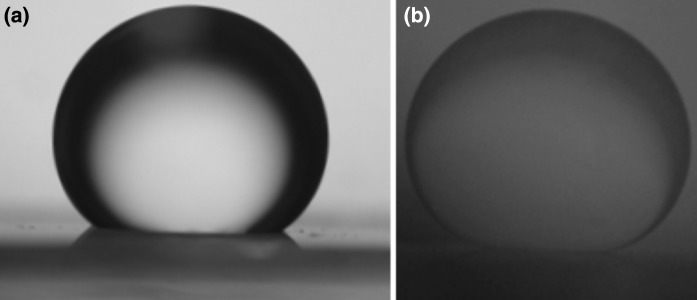
Decane droplet on quartz plate immersed in **a** water and **b** 75 nm quat/PEG (50:50)-modified silica nanoparticle dispersion of 0.5 wt%. The contact angle is **a** 35° and b 26°

**Fig. 21:**
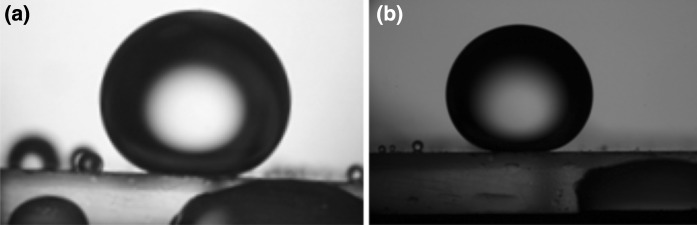
Decane droplet on quartz plate immersed in **a** water and **b** 5 nm PEG-modified silica nanoparticle dispersion of 1 wt%. The contact angle is **a** 20° and **b** 14°. The quartz plate in **a** is not tilted

**Fig. 22:**
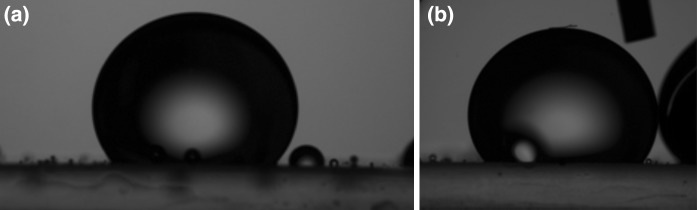
Decane droplet on quartz plate immersed in **a** water and **b** 25 nm PEG-modified silica nanoparticle dispersion of 1 wt%. The contact angle is **a** 56° and **b** 51°

**Fig. 23:**
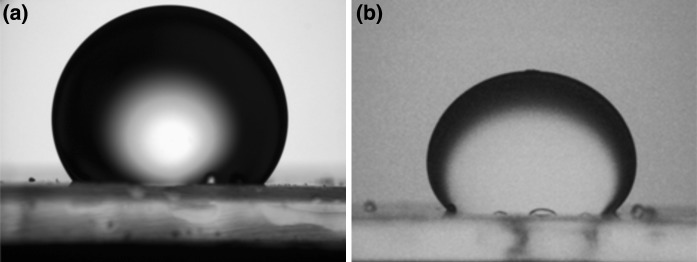
Decane droplet on quartz plate immersed in **a** water and **b** 75 nm PEG-modified silica nanoparticle dispersion of 0.5 wt%. The contact angle is **a** 52° and **b** 49°. The *color* in **b** is digitally altered to see the droplet clearly since the light transmittance of nanoparticle dispersion is low because of particle size. (Color figure online)

**Fig. 24:**
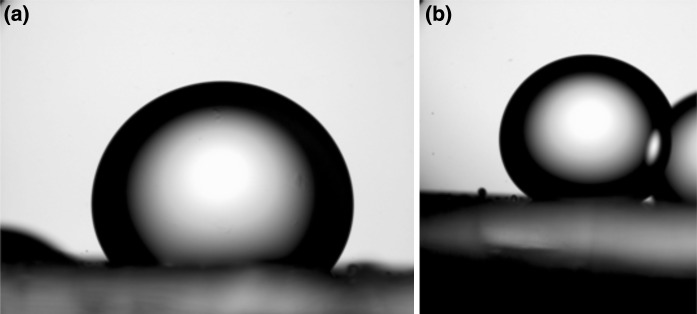
Decane droplet on quartz plate immersed in **a** water at 80 °C and **b** 5 nm PEG-modified silica nanoparticle dispersion of 1 wt% at 80 °C. The contact angle is **a** 59° and **b** 45°

**Fig. 25:**
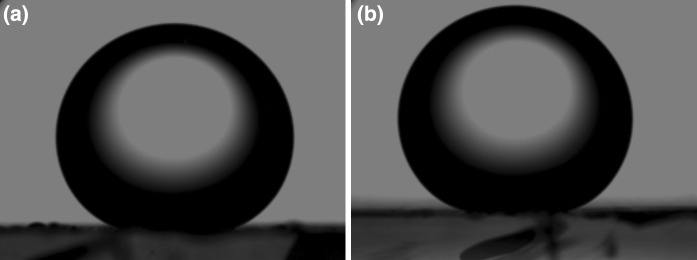
Decane droplet on calcite plate immersed in **a** water and **b** 5 nm sulfonate-modified silica nanoparticle dispersion of 1 wt%. The contact angle is **a** 32° and **b** 24°

**Fig. 26:**
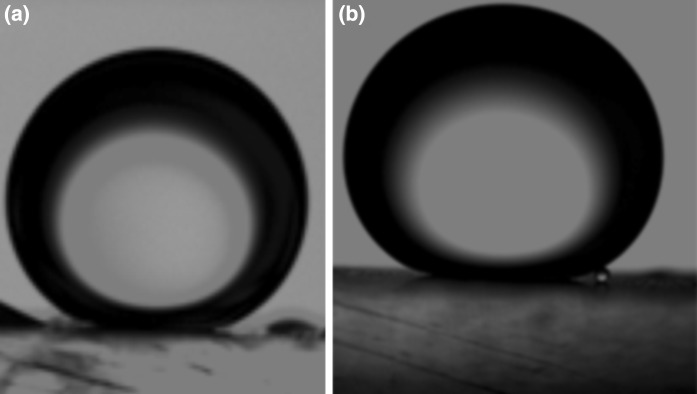
Decane droplet on calcite plate immersed in **a** water and **b** 25 nm sulfonate-modified silica nanoparticle dispersion of 1 wt%. The contact angle is **a** 21° and **b** 18°

**Fig. 27:**
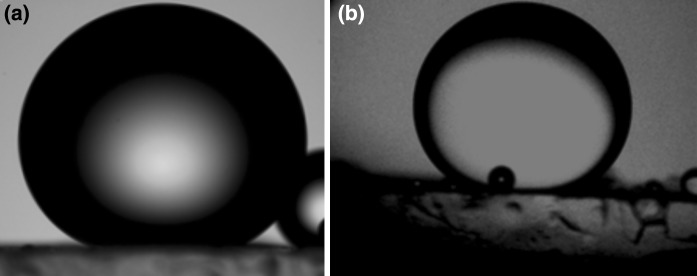
Decane droplet on calcite plate immersed in **a** water and **b** 75 nm sulfonate-modified silica nanoparticle dispersion of 0.5 wt%. The contact angle is **a** 38° and **b** 36°

A summary of contact angle measurements is presented in Fig. 14. The change in contact angle in the presence of nanoparticles is plotted as a function of nanoparticle diameter. The change is less than 104°.

## Conclusions

Significant adsorption of unmodified, sulfonate, or PEG-modified silica nanoparticles on quartz and calcite surfaces is not observed under the experimental conditions reported in this paper. Increase in particle size from 5 to 25 nm or addition of NaCl less than CSC does not promote adsorption of nanoparticles on mineral surfaces.

Unmodified nanoparticles or those with an anionic (sulfonate) or cationic surfactant (quat) do not influence the IFT of water/decane interface. The particle size or concentration does not have any influence on IFT. However, the presence of PEG as a surface coating material significantly decreases the IFT. The degree of change is the same for aqueous solutions of surface modifying materials in the absence of nanoparticles. Based on these results, it can be concluded that silica nanoparticles are not amphiphiles. The surface modification determines the extent of adsorption of silica particles to interfaces.

A slight change in contact angle is observed in the presence of unmodified or surface-modified nanoparticles with anionic, cationic or nonionic surfactants (sulfonate, quat, or PEG). The size of nanoparticle does not influence contact angle.

We further the study of Wasan and Nikolov (2003), Binks and Whitby (2005) and Lee et al. (2006) and investigate the effect of nanoparticles and surface treatment on IFT, adsorption on minerals and finally on contact angle change. We show that surface-modified silica nanoparticles have minimal interaction with minerals and the water/decane interface and hence the change in contact angle is not significant. We isolate the effect of surface treatment on the IFT change and conclude that the type and amount of surface treatment attached to silica nanoparticles determines the extent of the change in IFT of water/decane interface.

## Appendix

See Figs. 15, 16, 17, 18, 19, 20, 21, 22, 23, 24, 25, 26, and 27.
